# Hepatitis A outbreak associated with food consumption and sexual practices: a case-control study, Curitiba, Paraná, 2024

**DOI:** 10.1590/S2237-96222026v35e20250867.en

**Published:** 2026-03-16

**Authors:** Janilza Silveira Silva, Renata Barbosa Vilaça Marques de Carvalho, Alcides Souto de Oliveira, Liza Regina Bueno Rosso, Diego Spinoza dos Santos, Leia Regina Silva, Cláudia Weingaertner Palm, Clea Elisa Ribeiro, Katiuska Ferraz Jansen Negrello, Dirlene Pacheco Venâncio, Monique Boese, Aroldo José Borges Carneiro, Tatiane Bartneck Telles, Silvio Luis Rodrigues Almeida

**Affiliations:** 1 Ministério da Saúde, Programa de Treinamento em Epidemiologia Aplicada aos Serviços do SUS, Brasília, DF, Brazil; 2 Centro de Epidemiologia da Secretaria Municipal da Saúde de Curitiba, Paraná, PR, Brazil; 3 Centro de Informações Estratégicas em Vigilância em Saúde de Curitiba, Paraná, PR, Brazil; 4 Prefeitura de Curitiba, Secretaria Municipal da Saúde, Curitiba, Paraná, PR, Brazil

**Keywords:** Hepatitis A Virus, Disease Outbreaks, Foodborne Diseases, Sexual Behavior, Case-Control Studies, Virus de la Hepatitis A, Brotes de Enfermedades, Enfermedades Transmitidas por los Alimentos, Comportamiento Sexual, Estudios de Casos y Controles

## Abstract

**Objective:**

To identify factors associated with hepatitis A infection in Curitiba, Paraná, Brazil.

**Methods:**

This is a case-control study with individuals aged ≥16 years, living in Curitiba, Paraná, selected from the municipal laboratory base. We defined as cases individuals with reactive serology for hepatitis A and as controls those with non-reactive serology, from November 1^st^, 2023 to May 29^th^, 2024. Data were collected using a self-administered questionnaire. Bivariate analysis was performed followed by hierarchical logistic regression to estimate odds ratio (OR) and respective 95% confidence intervals (95%CI).

**Results:**

A total of 242 individuals participated (121 cases and 121 controls). The cases had a higher proportion of males (64.5% vs. 52.5%), Whites (78.5% vs. 63.8%), and men who have sex with men (MSM) (25.6% vs. 12.4%) when compared to controls. In the adjusted multivariate model, hepatitis A infection maintained a significant association with raw fish consumption (aOR 2.54; 95%CI 1.39; 4,64; p-value 0.002) and with MSM (aOR 2.38; 95%CI 1.10; 4,85; p-value 0.027). Not eating out had a protective effect (aOR 0.22; 95%CI 0.08; 0,62; p-value 0.004).

**Conclusion:**

In the context of the investigated outbreak, hepatitis A infection was associated with the consumption of raw fish and identification as MSM, while not eating out was a protective factor. These findings indicate the relevance of prevention measures related to both food security and specific population groups, especially in scenarios of urban outbreaks.

## Resumen

**Objetivos:**

Identificar los factores asociados a la infección por hepatitis A en Curitiba, Paraná (Brasil).

**Métodos:**

Se trata de un estudio de caso-control con individuos ≥16 años de edad, residentes en Curitiba, Paraná, seleccionados de la base del laboratorio municipal. Definimos como casos a individuos con serología reactiva para hepatitis A y como controles aquellos con serología no reactiva, en el periodo del 1/11/2023 al 29/5/2024. Los datos se recopilaron mediante un cuestionario autoadministrado. Se realizó un análisis bivariado, seguido de una regresión logística jerárquica, para estimar odds ratios (OR) y los respectivos intervalos de confianza del 95% (IC 95%).

**Resultados:**

Participaron 242 individuos (121 casos y 121 controles). Los casos mostraron una mayor proporción de individuos del sexo masculino (64,5% *versus* 52,5%), blancos (78,5% *vs.* 63,8%) y de hombres que tienen sexo con hombres (HSH) (25,6% *vs.* 12,4%) en comparación con los controles. En el modelo multivariado ajustado, la infección por hepatitis A mantuvo una significativa asociación con el consumo de pescado crudo (ORa 2,54; IC 95% 1,39; 4,64; p-valor 0,002) y con ser HSH (ORa 2,38; IC 95% 1,10; 4,85; p-valor 0,027). No comer fuera de casa tuvo un efecto protector (ORa 0,22; IC 95% 0,08; 0,62; p-valor 0,004).

**Conclusión:**

En el contexto del brote investigado, la infección por hepatitis A se asoció con el consumo de pescado crudo y con la identificación como HSH, mientras que no comer fuera de casa demostró ser un factor protector. Estos hallazgos indican la relevancia de las medidas de prevención relacionadas tanto con la seguridad alimentaria como con grupos poblacionales específicos, especialmente en escenarios de brotes urbanos.

**Ethical aspects**

This research respected the ethical principles, having obtained the following approval data:

Research Ethics Committee: National Research Ethics Committee

Opinion number: 7.545.013

Approval date: 13/6/2025

Certificate of Submission to Ethical Appraisal: 87956525.0.0000.0008

Informed consent: Dismissed.

## Introduction

Hepatitis A is an acute viral infection caused by the Hepatitis A Virus (HAV), with predominantly fecal-oral transmission ^1^. Very rarely cases of transmission of HAV via transfusion of blood products have been reported ^2^ ^,^ ^3^. Although it is historically related to poor basic sanitation conditions, outbreaks in urban environments have occurred with increasing frequency, also affecting populations with greater access to health services ^4^ ^-^ ^6^. This change in the epidemiological pattern reflects the transition from endemic circulation to punctual outbreaks, in which infection occurs in susceptible individuals ^7^ ^,^ ^8^, particularly young adults, with the potential for greater clinical severity ^1^.

The average incubation period for hepatitis A is 28 days, ranging from 15 to 50 days ^1^. Infected people can transmit the virus from the incubation period until about a week after the onset of jaundice ^9^, which can lead to the prolongation of outbreaks for long periods.

Transmission can occur via contaminated water or food, especially products consumed raw or poorly sanitized ^10^. Food outbreaks related to the consumption of contaminated raw fish, shellfish, or fruits have been described in several countries ^4^ ^,^ ^11^ ^-^ ^14^. Moreover, there is consistent evidence of the relevance of sexual practices as a route of infection, especially among men who have sex with men (MSM), with reports of outbreaks in large urban centers in Europe and the Americas ^15^ ^-^ ^20^. In Brazil, recent outbreaks of hepatitis A have been recorded in São Paulo (2017), Florianópolis (2023), and Campo Grande (2024) ^8^ ^,^ ^20^ ^,^ ^21^, including outbreak with reported person-to-person transmission ^20^. 

This study aimed to identify factors associated with hepatitis A infection in Curitiba, Paraná, Brazil.

## Methods

### Study design

This is an unmatched case-control study with participants living in Curitiba, state of Paraná, Brazil, who underwent a diagnostic test for Hepatitis A Virus (HAV) at the Municipal Laboratory of Curitiba. 

### Context

In 2023, Curitiba, Paraná, faced an outbreak of hepatitis A, with no clearly identified source of exposure. From November 1^st^, 2023 to May 29^th^, 2024, 281 cases were confirmed, of which five died and one case required liver transplantation. Infection occurred predominantly in young adult males ^22^. In view of this scenario, a team from the Training Program in Epidemiology Applied to the Services of the Unified Health System (EpiSUS-Avançado), in partnership with the local surveillance team, conducted this investigation. 

### Participants

Residents in the municipality of Curitiba who underwent a diagnostic test for hepatitis A in the Laboratory of Curitiba between November 1^st^, 2023 and May 29^th^, 2024 were eligible. 

We defined cases as individuals aged ≥16 years, residing in Curitiba, with a reactive result for antibodies against the Hepatitis A Virus - Immunoglobulin M (anti-HAV IgM), between November 1^st^, 2023 and May 29^th^, 2024. Controls were defined as individuals aged ≥16 years, living in the municipality, with non-reactive results for anti-HAV IgM, in the same period and laboratory. 

For participants selection, we adopted a 1:1 case-control ratio, without matching. All cases diagnosed during the study period were invited to participate. We selected the controls by random sampling, based on the list of all individuals who underwent testing at the Laboratory of Curitiba and had a non-reactive result for anti-HAV IgM. Initially, the number of controls equivalent to the number of eligible cases was drawn (one control for each case) and additionally a reserve of 33% of controls was selected for replacement, in case the minimum number was not reached.

In both groups, we excluded individuals with impaired cognitive ability, as informed by a family member or legal guardian. Among the controls, those who tested positive in another anti-HAV IgM serological test performed during the study period were also excluded.

### Variables

The study variables were defined based on a review of the scientific literature and preliminary data from the epidemiological investigation, namely:

- Sociodemographic: gender, age, race/skin-color, sexual orientation, schooling level, and income;
- Food history: places of purchase of food for home, eating out or ordering food, consumption of specific foods, origin of water for consumption, forms of water treatment for consumption, occurrence of interruptions or works in the water supply at home, interruption/work in the neighborhood’s sewage network;
- Personal hygiene: frequency of handwashing, use of soap in handwashing, handwashing after using the toilet, handwashing before preparing or consuming food, sharing personal objects, sanitizing fruits and vegetables;
- Participation in aquatic events or activities: attending swimming pools, clubs or water parks, and saunas; participation in parties/events;
- Use of recreational substances: alcohol abuse, use of illicit drugs;
- Sexual behavior: intimate or sexual contact, sex of partners, number of partners, use of condoms, practice of oro-anal sex, anal penetration, use of condoms in anal penetration, anal penetration with objects, anal penetration with fingers, attendance at sex parties or similar establishments, identification as MSM;
- Travel history: trips outside the metropolitan area of Curitiba, the destination of the trip.

### Data Source

The researchers collected data via a self-administered online questionnaire, developed and made available on the REDCap platform. The link to access the cases and controls was sent via three strategies: (i) the “Saúde Já Curitiba” app; (ii) messages via WhatsApp, with resubmission at different times to reinforce adherence; (iii) telephone call, for non-respondents, with the possibility of resending the link by another channel. Reservation controls were addressed according to a specific schedule, considering the reduced collection time imposed by the outbreak. Data collection occurred from April 10^th^ to May 3^rd^, 2024.

### Biases

Given some sensitive questions, we considered the existence of potential social desirability and information biases, which the investigators tried to minimize by using a self-administered online questionnaire in order to ensure information anonymity. Likewise, we also considered a possible selection bias due to the number of participant losses both due to difficulty in contact due to outdated/incorrect registration data, and resistance from individuals who had already participated in other surveillance actions. To minimize this bias, we sought contact with the participants via different means of communication and moments in order to ensure knowledge of the survey and reinforce the request for participation. Moreover, strategies were also adopted to control confounding in the analytical stage, by directed acyclic graphs (DAG) and multivariate analysis with logistic regression.

### Study size

The study size was defined based on the sample available in the period investigated, including all confirmed cases of hepatitis A. No formal sample size was calculated, and a case-control ratio of 1:1 was adopted.

### Statistical analyses

Measures of absolute and relative frequency were calculated, as well as measures of central tendency and dispersion. 

The analysis of the associated factors occurred in two stages: a bivariate analysis followed by a multivariate analysis. In the bivariate stage, binary logistic regression was used to evaluate the association between each independent variable and the outcome, with an estimate of the odds ratio (OR) and respective 95% confidence intervals (95%CI). The variables that showed a statistically significant association (p-value <0.05) were considered candidates for the multivariate stage.

To build the multivariate model, the selected variables were organized based on a conceptual model represented by a DAG, considering possible confounders. Initially, the association between the independent variables was evaluated using the Fisher’s exact test. For those associated with each other, the association with the outcome was evaluated by logistic regression. The variables that remained associated with the outcome (p-value <0.05) were included in a hierarchical logistic regression model. The term “hierarchical” refers to the sequential entry of variables into previously defined conceptual blocks.

The model was structured in three blocks: in the first, distal variables related to food and beverage consumption were included; in the second, an intermediate variable of a behavioral nature, a potential mediator of the relationship between eating habits and the outcome; and, in the third, a proximal variable, considered to be more closely related to the occurrence of hepatitis A. Insertion order was based on conceptual plausibility, the characteristics observed in the investigated outbreak and evidence of recent outbreaks described in the national and international literature. This approach allowed us to progressively evaluate the contribution of each set of variables and control for potential confounding factors at different levels.

A significance level of α<0.05 was adopted. The initial tabulation was carried out in Microsoft Excel 2016 and the statistical analysis in SPSS (v. 25) and RStudio (v. 4.4.0).

## Results

**Figure 1:**
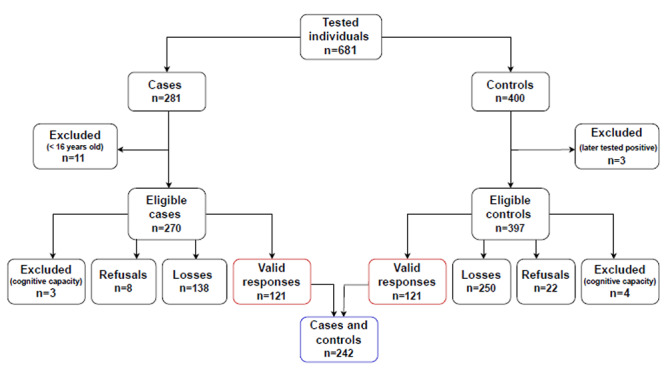
Selection of cases and controls in the hepatitis A study. Curitiba, Paraná, 2024 (n=242)

During the study period, 681 hepatitis A diagnosis tests were performed, with 281 reactive individuals, of which 270 were eligible. Of the 400 controls available for the same period, 397 were eligible. After considering refusals, exclusions and losses, 121 cases and 121 controls were recorded (Figure 1). 

There was a predominance of males (64.5%) and White (78.5%) among cases compared to controls. Regarding sexual orientation, most of the controls declared themselves heterosexual, while there was a higher proportion of homosexual or bisexual individuals among the cases (Table 1).

In the bivariate analysis, variables associated with hepatitis A virus infection included consumption of raw fish, grapes, raisins, sugarcane juice, eating out (in restaurants and sushi bars), recent sexual activity, being in the group of men who have sex with men (MSM), and attending sex parties. Not eating out was a protective factor (Table 2).

**Table 1:** Sociodemographic characteristics of participants in the hepatitis A case-control study. Curitiba, Paraná, 2024 (n=242)

Characteristic	Cases (%)	Controls (%)
Sex (%)	(n=121)	(n=120)
Male	78 (64.5)	63 (52.5)
Female	42 (34.7)	57 (47.5)
Intersex	1 (0.8)	0 (0.0)
Race/ethnicity	(n=121)	(n=116)
White	95 (78.5)	74 (63.8)
Mixed-race	21 (17.4)	35 (30.2)
Yellow	3 (2.5)	3 (2.6)
Black	2 (1.7)	3 (2.6)
Indigenous	0 (0.0)	1 (0.9)
Sexual orientation	(n=114)	(n=114)
Heterosexual	76 (66.7)	84 (73.7)
Homosexual	30 (26.3)	21 (18.4)
Bisexual	8 (7.0)	9 (7.9)
Age (years)	(n=121)	(n=120)
Mean (± Standard Deviation)	34.1 (± 8.5)	31.7 (± 9.3)
Income^b^	(n=117)	(n=110)
Up to 2 salaries (up to R$ 2,824.00)	27 (23.1)	38 (34.5)
From 2 to 5 salaries (R$ 2,824.01 to R$ 7,060.00)	55 (47.0)	49 (44.5)
From 5 to 10 salaries (R$ 7,060.01 to R$ 14,120.00)	20 (17.1)	14 (12.7)
More than 10 salaries (more than R$ 14,120.00)	15 (12.8)	5 (4.5)
No income	0 (0.0)	4 (3.6)
Schooling level	(n=121)	(n=120)
Low schooling level^c^	2 (1.7)	2 (1.7)
Middle/High School^d^	27 (22.3)	45 (37.5)
Higher education (incomplete or complete)	61 (50.4)	52 (43.3)
Graduate studies (incomplete or complete)	31 (25.6)	21 (17.5)

^a^ The values of ‘n’ in each variable vary according to the available data; ^b^Minimum wage in force in 2024: R$1,412.00; ^c^no schooling +incomplete middle school; ^d^complete middle school/incomplete or complete high school.

**Table 2:** Odds ratio (OR) and 95% confidence intervals (95%CI) of factors associated with hepatitis A. Curitiba, Paraná, 2024 (n=242)

Food^a^	Cases (%)	Controls (%)	OR	95%CI	p-value^b^
Raw fish (sushi/sashimi) (n=212)					
Yes	53 (51.96)	28 (25.45)	3.17	1.78; 5.65	<0.001
No	49 (48.04)	82 (74.55)			
Dried fruits (raisins) (n=208)					
Yes	36 (36.73)	23 (20.91)	2.19	1.19; 4.07	0.014
No	62 (63.27)	87 (79.09)			
Grapes (n=207)					
Yes	69 (68.32)	49 (46.23)	2.51	1.42; 4.42	0.002
No	32 (31.68)	57 (53.77)			
Sugarcane juice (n=205)					
Yes	51 (51.52)	39 (36.79)	1.82	1.04; 3.19	0.036
No	48 (48.48)	67 (63.21)			
Restaurant (n=236)					
Yes	81 (68.64)	56 (47.46)	2.42	1.42; 4.12	0.001
No	37 (31.36)	62 (52.54)			
Sushi bar (n=236)					
Yes	41 (34.75)	18 (15.25)	2.96	1.58; 5.55	0.001
No	77 (65.25)	100 (84.75)			
Did not eat out (n=236)					
Yes	5 (4.24)	29 (24.58)	0.14	0.05; 0.37	<0.001
No	113 (95.76)	89 (75.42)			
Delivery services (n=242)					
Yes	53 (43.8)	34 (28.1)	1.99	1.19; 3.40	0.016
No	68 (56.2)	87 (71.9)			
Sexual behavior (n=242)					
MSM^c^	31 (25.62)	15 (12.40)	2.43	1.23; 4.79	0.013
Not MSM	90 (74.38)	106 (87.60)			
Sexual intercourse in the last 50 days (n=226)					
Yes	99 (86.84)	84 (75.0)	2.20	1.10; 4.39	0.028
No	15 (13.16)	28 (25.0)			
Attending to sex parties (n=227)					
Yes	14 (12.28)	3 (2.65)	5.13	1.43; 18.39	0.010
No	100 (87.72)	110 (97.35)			

^a^ The values of ‘n’ in each variable vary according to the available data; ^b^Bivariate logistic regression; ^c^men who have sex with men.

**Figure 2:**
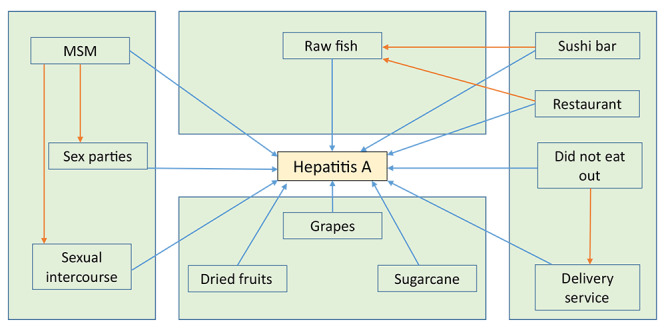
Variables associated with hepatitis A in the bivariate analysis (blue arrows) and potential confounding relationships considered in the hierarchical model (orange arrows), hepatitis A case-control study. Curitiba, Paraná, 2024 (n=242)

The variables associated with the outcome in the bivariate analysis were organized in a DAG for confounding assessment (Figure 2). For all pairs of variables for which confounding was considered, the association between them was tested using Fisher’s exact test, followed by bivariate logistic regression to test the relationship with the outcome. Thus, the variables “being MSM” (OR 2.09; 95%CI 1.04; 4.23; p-value 0.039), “eat raw fish” (OR 2.56; 95%CI 1.39; 4.73; p-value 0.002) and “do not eat out” (OR 0.15; 95%CI 0.05; 0.42; p-value<0.001).

The statistically significant variables (p-value<0.05) resulting from the previous analysis (being MSM, having consumed raw fish, and not having eaten out), together with the variables in which no possible confounding was observed (having consumed sugarcane juice, grapes, and raisins), were then analyzed using hierarchical logistic regression, according to the established model. Thus, in the block of distal variables, having consumed “sugarcane juice,” “grapes,” “raisins,” and “raw fish” were included, while in the intermediate and proximal blocks, the variables “not eating out” and “being MSM,” respectively, were included. Thus, statistically significant associations were found, indicating that the cases had 2.38 (95%CI 1.10; 4.85) times the chance of being MSM, and 2.54 (95%CI 1.39; 4.64) times the chance of having consumed raw fish, when compared to controls. Nevertheless, individuals who did not eat out (OR 0.22; 95%CI 0.08; 0.62; p-value 0.004) had a 78% lower chance of contracting hepatitis A, when compared to those who ate in restaurants. The other variables in the distal block did not remain associated with the outcome after adjustment (Table 3). This model featured a R^2^ of 18.8%.

**Table 3:** Odds ratio (OR), adjusted odds ratio (aOR) and 95% confidence intervals (95%CI) of the variables associated with hepatitis A. Curitiba, Paraná, 2024 (n=242)

Section^a^	Parameter	OR^b^	ORa	95%CI	p-value^c^
Section 1	Grapes	2.50	1.69	0.85; 3.36	0.132
	Dried fruits (raisins)	2.19	1.42	0.67; 2.98	0.335
	Sugarcane juice	1.82	1.34	0.70; 2.53	0.375
	Raw fish	3.17	2.55	1.34; 4.87	0.005
Section 2	Raw fish	3.17	2.56	1.41; 4.65	0.002
	Did not eat out	0.14	0.21	0.08; 0.58	0.003
Section 3	Raw fish	3.17	2.54	1.39; 4.64	0.002
	HSH^d^	2.43	2.38	1.10; 4.85	0.027
	Did not eat out	0.14	0.22	0.08; 0.62	0.004

^a^ The unadjusted OR refers to the bivariate analysis and remains constant in the different blocks of the hierarchical model; ^b^Section 1: Log Likelihood=231.77; R^2^ Nagelkerke=0.12; Hosmer and Lemeshow p-value=0.694. Section 2: Log Likelihood=231.77; R^2^ Nagelkerke=0.12; Hosmer and Lemeshow p-value=0.694. Section 3: Log Likelihood=261.32; R^2^ Nagelkerke=0.188; Hosmer and Lemeshow p-value= 0.792; ^c^Hierarchical logistic regression; ^d^men who have sex with men.

## Discussion

This study findings show relevant associations between hepatitis A infection and specific eating habits and sexual behaviors in adults during the outbreak in Curitiba, Paraná. Raw fish consumption, eating out, and specific sexual practices, namely (MSM), remained associated with the outcome even after adjusting for potential confounders. These results reinforce the multifactorial nature of Hepatitis A virus transmission, involving both dietary and interpersonal transmission. 

The final model explained approximately 18.8% of the outcome variability. Although this percentage may seem modest, in Epidemiological and Social Science studies it is expected that measures of pseudo-R² will have reduced magnitudes, since the occurrence of infectious diseases results from the interaction of multiple biological, behavioral, and social factors, which are not always measurable. Thus, the result is considered adequate and reinforces the relevance of the significant variables identified, which, although they explain part of the variability, contribute to the understanding of the determinants of the outcome and provide important support for prevention and intervention strategies. The model evolved from a limited explanation in the first block to greater explanatory power in the third, maintaining adequate fit in all stages, which reinforces the relevance of determinants at different hierarchical levels for the occurrence of hepatitis A. These findings suggest that, although distal factors related to food consumption initially contributed to explain part of the risk of hepatitis A, the inclusion of the intermediate variable (“not eating out”) and, above all, the proximal variable (“being MSM”) expanded the predictive capacity of the model, reinforcing the importance of considering different levels of determination in understanding the occurrence of the disease.

The association with raw fish consumption is consistent with the global literature that indicates raw or insufficiently cooked seafood as vehicles for the hepatitis A virus, highlighting the vulnerability to contamination during handling or at origin ^4^ ^,^ ^11^. By contrast, as not eating out proved to be a significant protective factor, it suggests that collective dining environments, including restaurants and delivery services, may have posed a substantial risk of exposure in the outbreak. This evidence reinforces the need for better health surveillance actions and improvement in good food handling practices in restaurants. Moreover, the significant association with self-identification as MSM underscores the importance of interpersonal transmission via sexual intercourse (oro-anal contact) in this group, a current epidemiological pattern that has been well documented in hepatitis A virus outbreaks globally ^15^ ^-^ ^20^. The confirmation of these routes of transmission in Curitiba, Paraná, is relevant, highlighting the need for comprehensive and adapted public health actions. These should go beyond traditional interventions focused only on sanitation and consider the multifaceted epidemiology of hepatitis A virus in contemporary urban outbreaks. 

Although the specific source of initial contamination was not identified, the findings make relevant contributions to the epidemiological knowledge of hepatitis A. The adjusted analysis strengthens the evidence that both dietary exposure and sexual behaviors played a role in the outbreak. Moreover, the data contribute to the understanding of at-risk population profiles and to the targeting of specific prevention actions, including expanding vaccination in vulnerable populations. 

Despite these important contributions, this study has limitations. There may have been a selection bias due to the number of losses of participants, both due to difficulty in contact due to outdated/incorrect registration data, and resistance from individuals who had already participated in other surveillance actions. This situation could either overestimate or underestimate the associations found. However, due to the lack of knowledge of some behavioral characteristics and social habits of the participants, it is not possible to adequately estimate the impact of this bias in this study, although they corroborate findings about associated factors in similar studies. Moreover, the questionnaire included sensitive questions, raising possible social desirability bias, which could underestimate the observed chances of each associated factor. However, information anonymity was guaranteed as a way to circumvent this bias. Finally, the missing data on some untreated variables, and therefore were not included in the analysis, may have impacted the study power due to the decrease in sample size, thus leading to imprecision of odds ratio and the increase in the confidence interval. However, it is noteworthy that confidence intervals are not so wide, which leads us to believe that the impact was not so important as to interfere with the study outcome. Moreover, it was decided not to conduct imputation due to the exploratory nature and the context of the outbreak.

In summary, this study identified raw fish consumption, eating out of home, and sexual practices among MSM as factors significantly associated with hepatitis A infection in the outbreak in Curitiba, Paraná. While the findings provide evidence supporting the need for multifaceted public health actions to curb the spread of the hepatitis A virus, they should be interpreted in light of urban contexts with similar characteristics.

## Data Availability

The anonymized database used in this research is in the public domain in the SciELO Data repository of the journal Epidemiologia e Serviços de Saúde (RESS) available for consultation at https://doi.org/10.48331/SCIELODATA.BXNNLM. The use of this data requires the proper citation of the reference provided on the platform.
