# Supplementary material for: Hepatitis A outbreak associated with food consumption and sexual practices: a case-control study, Curitiba, Paraná, 2024
Source: Epidemiol Serv Saude. 2026 Mar 16;35:e20250867. doi: 10.1590/S2237-96222026v35e20250867.en (PMC12995281; doi:10.1590/S2237-96222026v35e20250867.en)
Supplement: Translation

# Translation: Surto de hepatite A associado ao consumo de alimentos e práticas sexuais: estudo caso-controle, Curitiba, Paraná, 2024

**DOI:** 10.1590/S2237-96222026v35e20250867.pt

**Authors:** Janilza Silveira Silva, Renata Barbosa Vilaça Marques de Carvalho, Alcides Souto de Oliveira, Liza Regina Bueno Rosso, Diego Spinoza dos Santos, Leia Regina Silva, Cláudia Weingaertner Palm, Clea Elisa Ribeiro, Katiuska Ferraz Jansen Negrello, Dirlene Pacheco Venâncio, Monique Boese, Aroldo José Borges Carneiro, Tatiane Bartneck Telles, Silvio Luis Rodrigues Almeida

**Affiliations:** 1 Ministério da Saúde, Programa de Treinamento em Epidemiologia Aplicada aos Serviços do SUS, Brasília, DF, Brasil; 2 Centro de Epidemiologia da Secretaria Municipal da Saúde de Curitiba, Paraná, PR, Brasil; 3 Centro de Informações Estratégicas em Vigilância em Saúde de Curitiba, Paraná, PR, Brasil; 4 Prefeitura de Curitiba, Secretaria Municipal da Saúde, Curitiba, Paraná, PR, Brasil

**Keywords:** Vírus da Hepatite A, Surtos de Doenças, Doenças Transmitidas por Alimentos, Comportamento Sexual, Estudos de Casos e Controles

## Abstract

**Objetivo:**

Identificar fatores associados à infecção por hepatite A em Curitiba, Paraná.

**Métodos:**

Trata-se de um estudo caso-controle com indivíduos de idade ≥16 anos, residentes em Curitiba, Paraná, selecionados a partir da base do laboratório municipal. Definimos como casos os indivíduos com sorologia reagente para hepatite A e como controles aqueles com sorologia não reagente, no período de 1/11/2023 a 29/5/2024. Os dados foram coletados por questionário autopreenchível. Realizou-se análise bivariada seguida de regressão logística hierárquica para estimar *odds ratio* (OR) e respectivos intervalos de confiança de 95% (IC95%).

**Resultados:**

Participaram 242 indivíduos (121 casos e 121 controles). Os casos apresentaram maior proporção de indivíduos do sexo masculino (64,5% versus 52,5%), brancos (78,5% vs. 63,8%) e de homens que fazem sexo com homens (HSH) (25,6% vs. 12,4%) quando comparados aos controles. No modelo multivariado ajustado, a infecção por hepatite A manteve associação significativa com o consumo de peixe cru (ORa 2,54; IC95% 1,39; 4,64; p-valor 0,002) e com ser HSH (ORa 2,38; IC95% 1,10; 4,85; p-valor 0,027). A não realização de refeições fora do domicílio apresentou efeito protetor (ORa 0,22; IC95% 0,08; 0,62; p-valor 0,004).

**Conclusão:**

No contexto do surto investigado, a infecção por hepatite A esteve associada ao consumo de peixe cru e à identificação como HSH, enquanto não realizar refeições fora do domicílio mostrou-se fator protetor. Esses achados indicam a relevância de medidas de prevenção relacionadas tanto à segurança alimentar quanto a grupos populacionais específicos, especialmente em cenários de surtos urbanos.

**Aspectos éticos**

Esta pesquisa respeitou os princípios éticos, obtendo os seguintes dados de aprovação:

Comitê de ética em pesquisa: Comissão Nacional de Ética em Pesquisa

Número do parecer: 7.545.013

Data de aprovação: 13/6/2025

Certificado de apresentação de apreciação ética: 87956525.0.0000.0008

Registro do consentimento livre e esclarecido: Dispensado.

## Introdução

A hepatite A é uma infecção viral aguda causada pelo Vírus da Hepatite A (HAV), com transmissão predominantemente fecal-oral ^1^. Muito raramente foram reportados casos de transmissão do HAV através de transfusão de produtos sanguíneos ^2^ ^,^ ^3^. Embora esteja relacionada historicamente a condições precárias de saneamento básico, surtos em ambientes urbanos têm ocorrido com crescente frequência, afetando também populações com maior acesso a serviços de saúde ^4^ ^-^ ^6^. Essa mudança no padrão epidemiológico reflete a transição da circulação endêmica para surtos pontuais, nos quais a infecção ocorre em indivíduos suscetíveis ^7^ ^,^ ^8^, particularmente jovens adultos, com potencial de maior gravidade clínica ^1^.

O período de incubação médio da hepatite A é de 28 dias, variando de 15 a 50 dias ^1^. Pessoas infetadas podem transmitir o vírus desde o período de incubação até cerca de uma semana após o aparecimento da icterícia ^9^, o que pode propiciar o prolongamento de surtos por longos períodos.

A transmissão pode ocorrer por meio de água ou alimentos contaminados, especialmente produtos consumidos crus ou mal higienizados ^10^. Surtos alimentares relacionados ao consumo de peixe cru, mariscos ou frutas contaminadas têm sido descritos em diversos países ^4^ ^,^ ^11^ ^-^ ^14^. Além disso, há evidências consistentes da relevância de práticas sexuais como via de infecção, especialmente entre homens que fazem sexo com homens (HSH), com relatos de surtos em grandes centros urbanos da Europa e das Américas ^15^ ^-^ ^20^. No Brasil, surtos recentes de hepatite A foram registrados em São Paulo (2017), Florianópolis (2023) e Campo Grande (2024) ^8^ ^,^ ^20^ ^,^ ^21^, incluindo surto com transmissão interpessoal relatada ^20^. 

Esta investigação teve como objetivo identificar fatores associados à infecção por hepatite A em Curitiba, Paraná.

## Métodos

### Delineamento do estudo

Trata-se de estudo caso-controle não pareado com participantes residentes em Curitiba, Paraná, Brasil, que realizaram teste diagnóstico para o Vírus da Hepatite A (HAV) no Laboratório Municipal de Curitiba (LMC). 

### Contexto

Em 2023, Curitiba, Paraná, enfrentou surto de hepatite A, sem fonte de exposição claramente identificada. No período de 1º de novembro de 2023 a 29 de maio de 2024 foram confirmados 281 casos, dos quais cinco vieram a óbito e um caso necessitou de transplante hepático. A infecção ocorreu predominantemente em indivíduos jovens adultos do sexo masculino ^22^. Diante desse cenário, uma equipe do Programa de Treinamento em Epidemiologia Aplicada aos Serviços do Sistema Único de Saúde (EpiSUS-Avançado), em parceria com a equipe de vigilância local, conduziu esta investigação. 

### Participantes

Foram elegíveis residentes no município de Curitiba que realizaram teste diagnóstico para hepatite A na rede do LMC entre 1º de novembro de 2023 e 29 de maio de 2024. 

Definimos como casos os indivíduos com idade ≥16 anos, residentes em Curitiba, com resultado reagente para anticorpos contra o Vírus da Hepatite A - Imunoglobulina M (anti-HAV IgM), entre 1º de novembro de 2023 e 29 de maio de 2024. Já os controles foram definidos como indivíduos com idade ≥16 anos, residentes no município, com resultado não reagente para anti-HAV IgM, no mesmo período e laboratório. 

Para a seleção de participantes, adotamos uma relação caso-controle de 1:1, sem pareamento. Todos os casos diagnosticados no período do estudo foram convidados a participar. Selecionamos os controles por amostragem aleatória simples, a partir da listagem de todos os indivíduos que realizaram exame no LMC e apresentaram resultado não reagente para anti-HAV IgM. Inicialmente sorteou-se um número de controles equivalente ao de casos elegíveis (1 controle para cada caso) e adicionalmente selecionou-se uma reserva de 33% de controles para reposição, caso não fosse alcançado o número mínimo estabelecido.

Em ambos os grupos, excluímos indivíduos com comprometimento da capacidade cognitiva, conforme informado por familiar ou responsável legal. Entre os controles, também foram excluídos aqueles que apresentaram resultado positivo em outro teste sorológico IgM anti-HAV realizado no período do estudo.

### Variáveis

As variáveis do estudo foram definidas com base em revisão da literatura científica e em dados preliminares da investigação epidemiológica. São elas:

- Sociodemográficas: sexo, idade, raça/cor, orientação sexual, escolaridade e renda;
- Histórico alimentar: locais de compra de alimentos para o domicílio, realização de refeições fora de casa ou por encomenda, consumo de alimentos específicos, origem da água para consumo, formas de tratamento da água para consumo, ocorrência de interrupções ou obras no abastecimento de água no domicílio, interrupção/obra na rede de esgoto do bairro;
- Práticas de higiene pessoal: frequência de lavagem das mãos, uso de sabão na lavagem das mãos, lavagem das mãos após uso do banheiro, lavagem das mãos antes de preparar ou consumir alimentos, compartilhamento de objetos pessoais, higienização de frutas e vegetais;
- Participação em eventos ou atividades aquáticas: frequentar piscinas, clubes ou parques aquáticos e saunas; participação em festas/eventos;
- Uso de substâncias recreativas: consumo abusivo de álcool, uso de drogas ilícitas;
- Hábitos sexuais: contato íntimo ou sexual, sexo dos parceiros, número de parceiros, uso de preservativo, prática de sexo oro-anal, penetração anal, uso de preservativos na penetração anal, penetração anal com objetos, penetração anal com dedos, frequência a festas com temáticas sexuais ou estabelecimentos similares, identificação como HSH;
- Histórico de viagens: viagens para fora da região metropolitana de Curitiba, destino da viagem.

### Fonte de dados

Os pesquisadores coletaram os dados por meio de questionário on-line autopreenchível, desenvolvido e disponibilizado na plataforma REDCap. Enviou-se o link de acesso aos casos e controles por três estratégias: (i) aplicativo “Saúde Já Curitiba”; (ii) mensagens via WhatsApp, com reenvio em diferentes momentos para reforço da adesão; (iii) chamada telefônica, para os não respondentes, com possibilidade de reenvio do link por canal de preferência. Os controles de reserva foram abordados conforme cronograma específico, considerando o tempo reduzido de coleta imposto pelo contexto do surto. A coleta de dados ocorreu entre 10 de junho e 3 de julho de 2024.

### Vieses

Dado a existência de perguntas sensíveis, consideramos a existência de um potencial viés de desejabilidade social e de informação, o qual os investigadores tentaram minimizar através da coleta de dados realizada por um questionário autopreenchível disponibilizado de forma on-line, de modo a garantir o sigilo das informações. Da mesma forma, também consideramos um possível viés de seleção em razão do número de perdas de participantes tanto por dificuldade de contato devido a dados cadastrais desatualizados/incorretos, como resistência de indivíduos que já haviam participado de outras ações de vigilância. A fim de minimizarmos esse viés, buscamos contato com os participantes por diferentes vias de comunicação e momentos com o intuito de garantir o conhecimento da ação e reforçar a solicitação de participação. Adicionalmente, adotaram-se estratégias também para controle de confundimento na etapa analítica, por meio da utilização de gráficos acíclicos direcionados (DAG) e de análise multivariada com regressão logística.

### Tamanho do estudo

O tamanho do estudo foi definido a partir da casuística disponível no período investigado, incluindo todos os casos confirmados de hepatite A. Não foi realizado cálculo formal de tamanho amostral, e adotou-se razão caso-controle de 1:1.

### Análises estatísticas

Calcularam-se medidas de frequência absoluta e relativa, bem como medidas de tendência central e dispersão. 

A análise dos fatores associados ocorreu em duas etapas: uma análise bivariada seguida de análise multivariada. Na etapa bivariada, foi utilizada regressão logística binária para avaliar a associação entre cada variável independente e o desfecho, com estimativa das *odds ratio* (OR) e respectivos intervalos de confiança de 95% (IC95%). As variáveis que apresentaram associação estatisticamente significativa (p-valor<0,05) foram consideradas candidatas para a etapa multivariada.

Para a construção do modelo multivariado, as variáveis selecionadas foram organizadas com base em um modelo conceitual representado por um DAG, considerando possíveis relações de confundimento. Inicialmente, avaliou-se a associação entre as variáveis independentes por meio do teste exato de Fisher. Para aquelas associadas entre si, procedeu-se à avaliação da associação com o desfecho por regressão logística. As variáveis que permaneceram associadas ao desfecho (p-valor<0,05) foram incluídas em um modelo de regressão logística hierárquica. O termo “hierárquica” refere-se à entrada sequencial das variáveis em blocos conceituais previamente definidos.

O modelo foi estruturado em três blocos: no primeiro, incluíram-se variáveis distais relacionadas ao consumo de alimentos e bebidas; no segundo, uma variável intermediária de natureza comportamental, potencial mediadora da relação entre hábitos alimentares e o desfecho; e, no terceiro, uma variável proximal, considerada de maior proximidade causal com a ocorrência da hepatite A. A ordem de inserção foi fundamentada em plausibilidade conceitual, nas características observadas no surto investigado e em evidências de surtos recentes descritos na literatura nacional e internacional. Essa abordagem permitiu avaliar progressivamente a contribuição de cada conjunto de variáveis e controlar potenciais fatores de confundimento em diferentes níveis.

Adotou-se um nível de significância de α<0,05. A tabulação inicial foi feita no Microsoft Excel 2016 e a análise estatística no SPSS (v. 25) e no RStudio (v. 4.4.0).

## Resultados

No período do estudo foram realizados um total de 681 exames para diagnóstico de hepatite A, com um total de 281 indivíduos reagentes. Destes, 270 eram elegíveis. Relativamente aos 400 controles disponíveis para o mesmo período, 397 eram elegíveis. Após considerar-se as recusas, exclusões e perdas, contabilizou-se 121 casos e 121 controles (Figura 1).

**Figura 1:** Seleção de casos e controles no estudo de hepatite A, Curitiba. Paraná, 2024 (n=242)

Observou-se predominância do sexo masculino (64,5%) e da raça/cor branca (78,5%) entre casos comparados aos controles. Quanto à orientação sexual, a maioria dos controles se declarou heterossexual, enquanto entre os casos houve maior proporção de indivíduos homossexuais ou bissexuais (Tabela 1).

Na análise bivariada, variáveis associadas à infecção pelo vírus da hepatite A incluíram o consumo de peixe cru, uva, passas, caldo de cana, realizar refeição fora de casa (em restaurantes e bares de sushi), atividade sexual recente, ser do grupo de homens que fazem sexo com homens (HSH) e frequentar festas temáticas sexuais. Não comer fora foi fator protetor (Tabela 2).

**Tabela 1:** Características sociodemográficas dos participantes do estudo caso-controle de hepatite A. Curitiba, Paraná, 2024 (n=242)

Variável^a^	Casos (%)	Controles (%)
Sexo (%)	(n=121)	(n=120)
Masculino	78 (64,5)	63 (52,5)
Feminino	42 (34,7)	57 (47,5)
Intersexo	1 (0,8)	0 (0,0)
Raça/cor da pele	(n=121)	(n=116)
Branca	95 (78,5)	74 (63,8)
Parda	21 (17,4)	35 (30,2)
Amarela	3 (2,5)	3 (2,6)
Preta	2 (1,7)	3 (2,6)
Indígena	0 (0,0)	1 (0,9)
Orientação sexual	(n=114)	(n=114)
Heterossexual	76 (66,7)	84 (73,7)
Homossexual	30 (26,3)	21 (18,4)
Bissexual	8 (7,0)	9 (7,9)
Idade (anos)	(n=121)	(n=120)
Média (± Desvio padrão)	34,1 (± 8,5)	31,7 (± 9,3)
Renda^b^	(n=117)	(n=110)
Até 2 salários (até R$ 2.824,00)	27 (23,1)	38 (34,5)
De 2 a 5 salários (de R$ 2.824,01 a R$ 7.060,00)	55 (47,0)	49 (44,5)
De 5 a 10 salários (de R$ 7.060,01 a R$ 14.120,00)	20 (17,1)	14 (12,7)
Mais de 10 salários (mais de R$ 14.120,00)	15 (12,8)	5 (4,5)
Sem renda	0 (0,0)	4 (3,6)
Escolaridade	(n=121)	(n=120)
Baixa escolaridade^c^	2 (1,7)	2 (1,7)
Fundamental/médio^d^	27 (22,3)	45 (37,5)
Ensino superior (incompleto ou completo)	61 (50,4)	52 (43,3)
Pós-graduação (incompleta ou completa)	31 (25,6)	21 (17,5)

^a^ Os valores de ‘n’ em cada variável variam de acordo com os dados disponíveis; ^b^Salário mínimo vigente em 2024: R$1.412,00; ^c^sem escolaridade + ensino fundamental/primário incompleto; ^d^ensino fundamental/primário completo + ensino médio incompleto/completo

**Tabela 2:** *Odds ratio* (OR) e intervalos de confiança de 95% (IC95%) dos fatores associados à hepatite A. Curitiba, Paraná, 2024 (n=242)

Alimentos^a^	Casos (%)	Controles (%)	OR	IC95%	p-valor^b^
*Peixe cru (sushi/sashimi) (n=212)*					
Sim	53 (51,96)	28 (25,45)	3,17	1,78; 5,65	<0,001
Não	49 (48,04)	82 (74,55)			
*Frutas secas (uva-passa) (n=208)*					
Sim	36 (36,73)	23 (20,91)	2,19	1,19; 4,07	0,014
Não	62 (63,27)	87 (79,09)			
*Uvas (n=207)*					
Sim	69 (68,32)	49 (46,23)	2,51	1,42; 4,42	0,002
Não	32 (31,68)	57 (53,77)			
*Caldo de cana (n=205)*					
Sim	51 (51,52)	39 (36,79)	1,82	1,04; 3,19	0,036
Não	48 (48,48)	67 (63,21)			
*Restaurante (n=236)*					
Sim	81 (68,64)	56 (47,46)	2,42	1,42; 4,12	0,001
Não	37 (31,36)	62 (52,54)			
*Sushi bar (n=236)*					
Sim	41 (34,75)	18 (15,25)	2,96	1,58; 5,55	0,001
Não	77 (65,25)	100 (84,75)			
*Não comeu fora (n=236)*					
Sim	5 (4,24)	29 (24,58)	0,14	0,05; 0,37	<0,001
Não	113 (95,76)	89 (75,42)			
*Serviços de entrega (n=242)*					
Sim	53 (43,8)	34 (28,1)	1,99	1,19; 3,40	0,016
Não	68 (56,2)	87 (71,9)			
*Hábito sexual (n=242)*					
HSH^c^	31 (25,62)	15 (12,40)	2,43	1,23; 4,79	0,013
Não HSH	90 (74,38)	106 (87,60)			
*Relação sexual nos últimos 50 dias (n=226)*					
Sim	99 (86,84)	84 (75,0)	2,20	1,10; 4,39	0,028
Não	15 (13,16)	28 (25,0)			
*Participação em festas temáticas sexuais (n=227)*					
Sim	14 (12,28)	3 (2,65)	5,13	1,43; 18,39	0,010
Não	100 (87,72)	110 (97,35)			

^a^ Os valores de n em cada variável variam de acordo com os dados disponíveis; ^b^Regressão logística bivariada; ^c^homens que fazem sexo com homens.

As variáveis associadas ao desfecho na análise bivariada foram organizadas em um gráfico acíclico direcionado para avaliação de confundimento (Figura 2). Para todas as duplas de variáveis para os quais se considerou haver confundimento, testou-se a associação entre elas através do teste exato de Fisher, seguido de regressão logística bivariada para testar a relação com o desfecho. Dessa forma, permaneceram associadas estatisticamente as variáveis “ser HSH” (OR 2,09; IC95% 1,04; 4,23; p-valor 0,039), “comer peixe cru” (OR 2,56; IC95% 1,39; 4,73; p-valor 0,002) e “não comer fora” (OR 0,15; IC95% 0,05; 0,42; p-valor<0,001).

**Figura 2:** Variáveis associadas à hepatite A na análise bivariada (setas azuis) e relações de potencial confundimento consideradas no modelo hierárquico (setas laranjas), estudo caso-controle de hepatite A. Curitiba, Paraná, 2024 (n=242)

As variáveis estatisticamente significativas (p-valor<0,05) resultantes da análise anterior (ser HSH, ter consumido peixe cru e não ter comido fora de casa), conjuntamente com as variáveis nas quais não se visualizou um possível confundimento (ter consumido caldo de cana, uvas e frutas secas) foram então analisadas através de regressão logística hierárquica, de acordo com o modelo estabelecido. Assim, no bloco de variáveis distais foram incluídas ter consumido “caldo de cana”, “uvas”, “frutas secas (uva-passa)” e “peixe cru”, enquanto nos blocos intermediários e proximais foram incluídas as variáveis “não comer fora” e “ser HSH”, respectivamente. Dessa forma, verificou-se associações estatisticamente significativas, indicando que os casos apresentaram 2,38 (IC95% 1,10; 4,85) vezes a chance de serem HSH, e 2,54 (IC95% 1,39; 4,64) vezes a chance de terem consumido peixe cru, quando comparados aos controles. Por outro lado, indivíduos que não comeram fora de casa (OR 0,22; IC95% 0,08; 0,62; p-valor 0,004) apresentaram uma chance 78% menor de contraírem hepatite A, quando comparados àqueles que comeram em restaurantes. As demais variáveis do bloco distal não permaneceram associadas ao desfecho após o ajuste (Tabela 3). O modelo final apresentou um-R^2^ de 18,8%.

**Tabela 3:** *Odds ratio* (OR), *odds ratio* ajustado (ORa) e intervalos de confiança de 95% (IC95%) das variáveis associadas à hepatite A. Curitiba, Paraná, 2024 (n=242)

Bloco ^a^	Variáveis	OR^b^	ORa	IC95%	p-valor^c^
Bloco 1	Uvas	2,50	1,69	0,85; 3,36	0,132
	Frutas secas (uva-passa)	2,19	1,42	0,67; 2,98	0,335
	Caldo de cana	1,82	1,34	0,70; 2,53	0,375
	Peixe cru	3,17	2,55	1,34; 4,87	0,005
Bloco 2	Peixe cru	3,17	2,56	1,41; 4,65	0,002
	Não comeu fora	0,14	0,21	0,08; 0,58	0,003
Bloco 3	Peixe cru	3,17	2,54	1,39; 4,64	0,002
	HSH^d^	2,43	2,38	1,10; 4,85	0,027
	Não comeu fora	0,14	0,22	0,08; 0,62	0,004

^a^ O OR bruto refere-se à análise bivariada e permanece constante nos diferentes blocos do modelo hierárquico; ^b^Bloco 1: Log Likelihood=231,77; R^2^ Nagelkerke=0,12; Hosmer e Lemeshow p-valor 0,694. Bloco 2: Log Likelihood=266,39; R^2^ Nagelkerke=0,161; Hosmer e Lemeshow p-valor= 0,781. Bloco 3: Log Likelihood=261,32; R^2^ Nagelkerke=0,188; Hosmer e Lemeshow p-valor 0,792; ^c^Regressão logística hierárquica; ^d^homens que fazem sexo com homens.

## Discussão

Os achados deste estudo evidenciam associações relevantes entre infecção pelo vírus da hepatite A e comportamentos alimentares e sexuais em adultos durante o surto ocorrido em Curitiba, Paraná. O consumo de peixe cru, o hábito de comer fora de casa e práticas sexuais específicas, nomeadamente homens que fazem sexo com homens (HSH), permaneceram associados ao desfecho mesmo após ajuste por potenciais fatores de confundimento. Esses resultados reforçam a natureza multifatorial da transmissão do Vírus da Hepatite A (HAV), envolvendo tanto vias alimentares quanto a transmissão interpessoal. 

O modelo final explicou aproximadamente 18,8% da variabilidade do desfecho. Embora esse percentual possa parecer modesto, em estudos epidemiológicos e de ciências sociais é esperado que medidas de pseudo-R² apresentem magnitudes reduzidas, uma vez que a ocorrência de doenças infecciosas resulta da interação de múltiplos fatores biológicos, comportamentais e sociais, nem sempre mensuráveis. Assim, o resultado encontrado é considerado adequado e reforça a relevância das variáveis significativas identificadas, que mesmo explicando parte da variabilidade, contribuem para a compreensão dos determinantes do desfecho e fornecem subsídios importantes para estratégias de prevenção e intervenção. O modelo evoluiu de explicação limitada no primeiro bloco para maior poder explicativo no terceiro, mantendo ajuste adequado em todas as etapas, o que reforça a relevância dos determinantes em diferentes níveis hierárquicos para a ocorrência da hepatite A. Esses achados sugerem que, embora os fatores distais relacionados ao consumo de alimentos tenham contribuído inicialmente para explicar parte do risco de hepatite A, a inclusão da variável intermediária (“Não comer fora”) e, sobretudo, da variável proximal (“Ser HSH”) ampliou a capacidade preditiva do modelo, reforçando a importância de considerar diferentes níveis de determinação na compreensão da ocorrência da doença.

A associação com o consumo de peixe cru é consistente com a literatura global que aponta produtos do mar crus ou insuficientemente cozidos como veículos do HAV, ressaltando a vulnerabilidade a contaminações durante o manuseio ou na origem ^4^ ^,^ ^11^. Em contrapartida, o fato de não comer fora de casa ter se revelado um fator protetor significativo sugere que ambientes de refeições coletivas, incluindo restaurantes e serviços de entrega, podem ter representado um risco substancial de exposição no surto. Essa evidência reforça a necessidade de ações de vigilância sanitária intensificada e melhoria nas boas práticas de manipulação de alimentos em estabelecimentos comerciais. Adicionalmente, a associação significativa com a autoidentificação como HSH sublinha a importância da transmissão interpessoal por via sexual (contato oro-anal) nesse grupo, um padrão epidemiológico atual e que tem sido bem documentado em surtos de HAV globalmente ^15^ ^-^ ^20^. A confirmação dessas vias de transmissão no contexto de Curitiba, Paraná, é relevante, o que evidencia a necessidade de ações de saúde pública abrangentes e adaptadas, que vão além das intervenções tradicionais focadas apenas em saneamento e consideram a multifacetada epidemiologia do HAV em surtos urbanos contemporâneos. 

Apesar de não se ter identificado a fonte de contaminação inicial específica, os achados trazem contribuições relevantes para o conhecimento epidemiológico da hepatite A. A análise ajustada fortalece a evidência de que tanto a exposição alimentar quanto os comportamentos sexuais desempenharam papel no surto ocorrido. Além disso, os dados contribuem para a compreensão de perfis populacionais em risco e para o direcionamento de ações de prevenção específicas, incluindo ampliação da vacinação em populações vulneráveis. 

Apesar dessas importantes contribuições, este estudo possui limitações. A coleta de dados em um período específico do surto pode não ter capturado todas as nuances da dinâmica de transmissão. Pode ter ocorrido um viés de seleção em razão do número de perdas de participantes tanto por dificuldade de contato devido a dados cadastrais desatualizados/incorretos, como resistência de indivíduos que já haviam participado de outras ações de vigilância. Essa situação poderia tanto superestimar quanto subestimar as associações encontradas. Entretanto, por desconhecer algumas características comportamentais e de hábitos sociais dos participantes, não é possível estimar adequadamente o impacto desse viés neste estudo, muito embora eles corroborem achados sobre fatores associados em estudos semelhantes. Adicionalmente, o questionário incluía questões sensíveis, o que levanta a possibilidade de viés de desejabilidade social, o qual poderia subestimar as chances observadas de cada fator associado. Contudo, garantiu-se o sigilo das informações como forma de contornar esse último viés. Por fim, a ocorrência de dados faltantes em algumas variáveis e que não foram tratados, e, portanto, não foram incluídos na análise, pode ter impactado o poder do estudo devido à diminuição do tamanho da amostra, acarretando, assim, imprecisão das medidas de associação (*odds ratio*) e do aumento do intervalo de confiança. Entretanto, os intervalos de confiança não são tão largos, o que nos leva a acreditar que o impacto não foi tão importante a ponto de interferir na conclusão do nosso estudo. Além disso, optou-se por não realizar imputação devido à natureza exploratória e ao contexto de surto.

Em suma, este estudo identificou o consumo de peixe cru, as refeições fora de casa e as práticas sexuais entre HSH como fatores significativamente associados à infecção por hepatite A no surto de Curitiba, Paraná. Embora os achados forneçam evidências que apoiam a necessidade de ações de saúde pública multifacetadas para conter a disseminação do HAV, eles devem ser interpretados à luz de contextos urbanos com características semelhantes.

## Data Availability

A base de dados anonimizada usada nesta investigação encontra-se em domínio público no repositório SciELO Data da revista *Epidemiologia e Serviços de Saúde* (RESS) disponível para consulta no endereço https://doi.org/10.48331/SCIELODATA.BXNNLM. A utilização desses dados requer a devida citação da referência fornecida na plataforma.
